# Pyrrolidinium-2-carboxyl­ate–4-nitro­phenol (1/2)

**DOI:** 10.1107/S1600536813028742

**Published:** 2013-10-31

**Authors:** Narayanan Swarna Sowmya, Yechuri Vidyalakshmi, Sadasivam Sampathkrishnan, Thothadri Srinivasan, Devadasan Velmurugan

**Affiliations:** a Sri Venkateswara College of Engineering, Pennalur, Irungattukottai 602 117, Sriperumbudur Taluk, Tamilnadu, India; b Department of Physics, Anna University, Adyar, Chennai 600 025, Tamilnadu, India; c Centre of Advanced Study in Crystallography and Biophysics, University of Madras, Guindy Campus, Chennai 600 025, India

## Abstract

In the title compound, C_5_H_9_NO_2_·2C_6_H_5_NO_3_, the pyrrolidine ring of the pyrrolidinium-2-carboxyl­ate zwitterion adopts a twisted conformation on the –CH_2_—CH_2_– bond adjacent to the N atom. The mean plane of this pyrrolidine ring forms dihedral angles of 25.3 (3) and 32.1 (3)° with the two nitro­phenol rings. An intra­molecular N—H⋯O hydrogen bond occurs in the pyrrolidinium-2-carboxyl­ate mol­ecule. In the crystal, mol­ecules are linked *via* O—H⋯O and N—H⋯O hydrogen bonds, enclosing *R* ^3^_2_(8) ring motifs, forming chains running parallel to the *a* axis. These chains are further cross-linked by O—H⋯O and C—H⋯O hydrogen bonds, forming undulating two-dimensional networks lying parallel to (001).

## Related literature

For the use of nitro-aromatics as inter­mediates in explosives, dyestuffs, pesticides and organic synthesis, see: Yan *et al.* (2006 ▶). For the occurrence of nitro-aromatics in industrial wastes and as direct pollutants in the environment, see: Yan *et al.* (2006 ▶); Soojhawon *et al.* (2005 ▶). For ring puckering analysis, see: Cremer & Pople (1975 ▶).
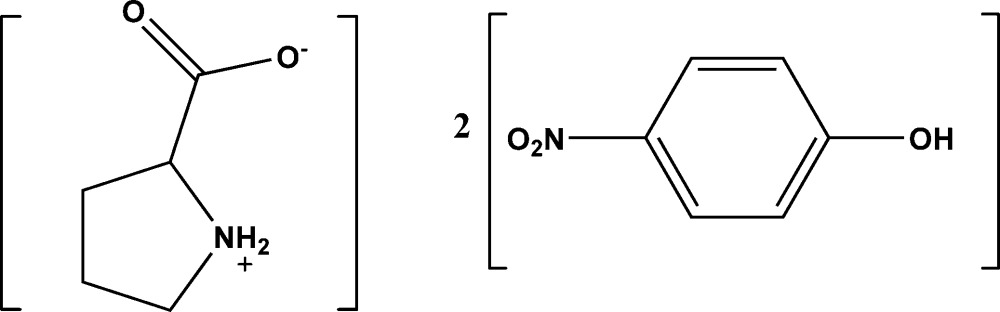


## Experimental

### 

#### Crystal data

C_5_H_9_NO_2_·2C_6_H_5_NO_3_
*M* *_r_* = 393.35
Orthorhombic, 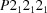

*a* = 5.9045 (3) Å
*b* = 15.6099 (7) Å
*c* = 20.0424 (9) Å
*V* = 1847.28 (15) Å^3^
*Z* = 4
Mo *K*α radiation
μ = 0.11 mm^−1^
*T* = 293 K
0.35 × 0.25 × 0.25 mm

#### Data collection

Bruker SMART APEXII area-detector diffractometer
Absorption correction: multi-scan (*SADABS*; Bruker, 2008 ▶) *T*_min_ = 0.961, *T*_max_ = 0.972
17765 measured reflections
3572 independent reflections
2987 reflections with *I* > 2σ(*I*)
*R*_int_ = 0.029

#### Refinement

*R*[*F* ^2^ > 2σ(*F* ^2^)] = 0.037
*wR*(*F* ^2^) = 0.094
*S* = 1.06
3572 reflections
261 parameters
2 restraints
H atoms treated by a mixture of independent and constrained refinement
Δρ_max_ = 0.23 e Å^−3^
Δρ_min_ = −0.15 e Å^−3^

### 

Data collection: *APEX2* (Bruker, 2008 ▶); cell refinement: *SAINT* (Bruker, 2008 ▶); data reduction: *SAINT*; program(s) used to solve structure: *SHELXS97* (Sheldrick, 2008 ▶); program(s) used to refine structure: *SHELXL97* (Sheldrick, 2008 ▶); molecular graphics: *ORTEP-3 for Windows* (Farrugia, 2012 ▶); software used to prepare material for publication: *SHELXL97* and *PLATON* (Spek, 2009 ▶).

## Supplementary Material

Crystal structure: contains datablock(s) global, I. DOI: 10.1107/S1600536813028742/su2657sup1.cif

Structure factors: contains datablock(s) I. DOI: 10.1107/S1600536813028742/su2657Isup2.hkl

Supplementary material file. DOI: 10.1107/S1600536813028742/su2657Isup3.cml

Additional supplementary materials:  crystallographic information; 3D view; checkCIF report

## Figures and Tables

**Table 1:** Hydrogen-bond geometry (Å, °)

*D*—H⋯*A*	*D*—H	H⋯*A*	*D*⋯*A*	*D*—H⋯*A*
N3—H3*A*⋯O8	0.94 (6)	2.03 (6)	2.606 (6)	118 (5)
N3—H3*B*⋯O7^i^	0.93 (5)	1.87 (6)	2.766 (6)	160 (7)
O3—H3*C*⋯O7^i^	0.82	1.92	2.656 (5)	148
O6—H6*A*⋯O8^ii^	0.82	1.82	2.604 (5)	159
C11—H11⋯O1^i^	0.93	2.59	3.503 (8)	169

Symmetry codes: (i) 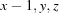; (ii) 
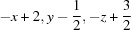
.

**Fig. 1.**
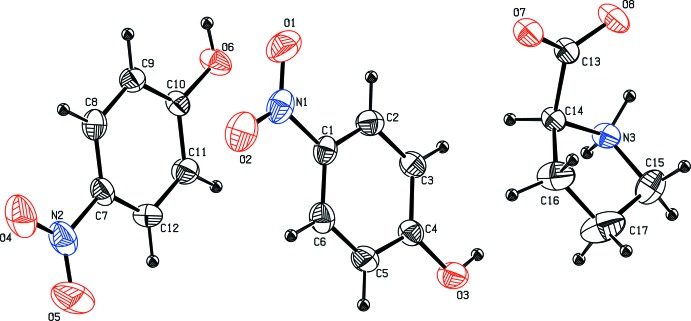
The molecular structure of the title compound, with atom labelling. Displacement ellipsoids are drawn at the 30% probability level.

**Fig. 2.**
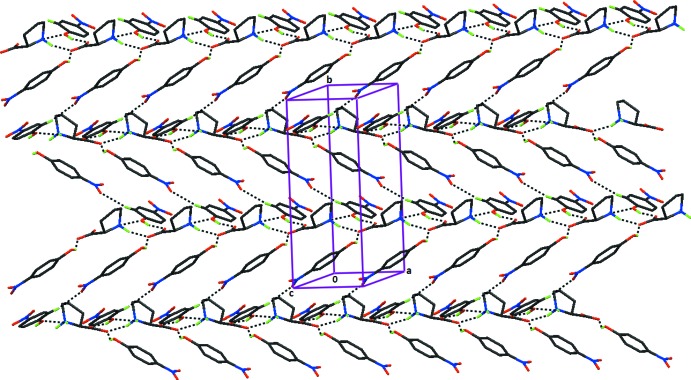
The crystal packing of the title compound viewed along the c axis. The hydrogen bonds are shown as dashed lines (see Table 1 for details; H atoms not involved in hydrogen bonding have been excluded for clarity).

## supplementary crystallographic information

### 1. Comment

Nitro-aromatics are widely used either as materials or as intermediates in explosives, dyestuffs, pesticides and organic synthesis (Yan *et al.*, 2006). They also occur as industrial wastes and direct pollutants in the environment. They are relatively soluble in water and detectable in rivers, ponds and soil (Yan *et al.*, 2006; Soojhawon *et al.*, 2005).

The title compound was synthesized by mixing eqimolar amounts of pyrrolidine carboxylic acid and para-nitrophenol in water. The crystals obtained were found to be composed of one molecule of pyrrolidinium-2-carboxylate, in the zwitterion form, and two molecules of para-nitrophenol, Fig. 1. The pyrrolidine ring (N3/C14-C17) adopts a twisted conformation on bond C17-C15, with puckering parameters (Cremer & Pople, 1975) q_2_ = 0.373 (7) Å and φ_2_ = 312.3 (11)°. The hydroxy group O atoms, O3 and O6, deviate slightly by -0.0380 (3) and 0.0160 (5) Å, respectively, from the mean planes of the benzene rings to which they are attached, (C1–C6) and (C7–C12).

The mean plane of the pyrrolidine ring (N3/C14-C17) forms dihedral angles of 25.3 (3)° and 32.1 (3)° with the nitro-phenol rings (C1-C6) and (C7-C12), respectively.

In the crystal, molecules are linked via O—H···O and N—H···O intra- and inter-molecular hydrogen bonds (Table 1 and Fig. 2), with R^3^_2_(8) ring motifs, forming chains running parallel to the a axis. These chains are further cross-linked by O—H···O and C—H···O hydrogen bond forming undulating two-dimensional networks lying parallel to the ab plane (Table 1 and Fig. 2).

### 2. Experimental

An equimolar (1:1:1) ratio of pyrrolidine carboxylic acid and para-nitrophenol were added to distilled water as solvent and the mixture stirred for 1 h, giving a clear solution. The solution was filtered into a clean beaker and sealed with parafilm and kept at room temperature for three days, after which block-like colourless crystals suitable for X-ray diffraction analysis were obtained.

### 3. Refinement

The NH H-atoms were located in difference electron-density maps and refined with distance restraints: N-H = 0.92 (2) Å. The OH and C-bound H-atoms were included in calculated positions and treated as riding atoms: O-H = 0.82 Å, C-H = 0.93, 0.97 and 0.98 Å for CH(aromatic), CH_2_, and CH(methine) H-atoms, respectively, with U_iso_(H) = 1.5U_eq_(C-methyl and O), and = 1.2U_eq_(C) for other H-atoms. In the final cycles of refinement, in the absence of significant anomalous scattering effects, 1490 Friedel pairs were merged and Δf '' set to zero.

### Crystal data

**Table:**

C_5_H_9_NO_2_·2C_6_H_5_NO_3_	*F*(000) = 824
*M**_r_* = 393.35	*D*_x_ = 1.414 Mg m^−^^3^
Orthorhombic, *P*2_1_2_1_2_1_	Mo *K*α radiation, λ = 0.71073 Å
Hall symbol: P 2ac 2ab	Cell parameters from 3572 reflections
*a* = 5.9045 (3) Å	θ = 2.4–25.9°
*b* = 15.6099 (7) Å	µ = 0.11 mm^−^^1^
*c* = 20.0424 (9) Å	*T* = 293 K
*V* = 1847.28 (15) Å^3^	Block, colourless
*Z* = 4	0.35 × 0.25 × 0.25 mm

### Data collection

**Table:**

Bruker SMART APEXII area-detector diffractometer	3572 independent reflections
Radiation source: fine-focus sealed tube	2987 reflections with *I* > 2σ(*I*)
Graphite monochromator	*R*_int_ = 0.029
ω and φ scans	θ_max_ = 25.9°, θ_min_ = 2.4°
Absorption correction: multi-scan (*SADABS*; Bruker, 2008)	*h* = −7→7
*T*_min_ = 0.961, *T*_max_ = 0.972	*k* = −17→19
17765 measured reflections	*l* = −23→24

### Refinement

**Table:**

Refinement on *F*^2^	Primary atom site location: structure-invariant direct methods
Least-squares matrix: full	Secondary atom site location: difference Fourier map
*R*[*F*^2^ > 2σ(*F*^2^)] = 0.037	Hydrogen site location: inferred from neighbouring sites
*wR*(*F*^2^) = 0.094	H atoms treated by a mixture of independent and constrained refinement
*S* = 1.06	*w* = 1/[σ^2^(*F*_o_^2^) + (0.0434*P*)^2^ + 0.2454*P*] where *P* = (*F*_o_^2^ + 2*F*_c_^2^)/3
3572 reflections	(Δ/σ)_max_ < 0.001
261 parameters	Δρ_max_ = 0.23 e Å^−^^3^
2 restraints	Δρ_min_ = −0.15 e Å^−^^3^

### Special details

**Table:**

Geometry. All esds (except the esd in the dihedral angle between two l.s. planes) are estimated using the full covariance matrix. The cell esds are taken into account individually in the estimation of esds in distances, angles and torsion angles; correlations between esds in cell parameters are only used when they are defined by crystal symmetry. An approximate (isotropic) treatment of cell esds is used for estimating esds involving l.s. planes.
Refinement. Refinement of F^2^ against ALL reflections. The weighted R-factor wR and goodness of fit S are based on F^2^, conventional R-factors R are based on F, with F set to zero for negative F^2^. The threshold expression of F^2^ > 2sigma(F^2^) is used only for calculating R-factors(gt) etc. and is not relevant to the choice of reflections for refinement. R-factors based on F^2^ are statistically about twice as large as those based on F, and R- factors based on ALL data will be even larger.

### Fractional atomic coordinates and isotropic or equivalent isotropic displacement parameters (Å^2^)

**Table:**

	*x*	*y*	*z*	*U*_iso_*/*U*_eq_	
C1	0.7666 (9)	0.5876 (3)	0.5668 (3)	0.0468 (13)	
C2	0.6897 (10)	0.5897 (3)	0.6313 (3)	0.0502 (13)	
H2	0.7673	0.5610	0.6649	0.060*	
C3	0.4946 (9)	0.6351 (3)	0.6455 (2)	0.0469 (12)	
H3	0.4380	0.6362	0.6887	0.056*	
C4	0.3835 (9)	0.6789 (3)	0.5951 (2)	0.0455 (12)	
C5	0.4649 (11)	0.6750 (4)	0.5306 (3)	0.0556 (15)	
H5	0.3892	0.7039	0.4967	0.067*	
C6	0.6561 (11)	0.6291 (4)	0.5163 (3)	0.0552 (14)	
H6	0.7103	0.6262	0.4728	0.066*	
C7	0.7088 (10)	0.3784 (3)	0.4091 (3)	0.0491 (13)	
C8	0.8784 (10)	0.3384 (4)	0.4433 (3)	0.0518 (14)	
H8	1.0042	0.3175	0.4207	0.062*	
C9	0.8621 (9)	0.3292 (3)	0.5114 (3)	0.0474 (12)	
H9	0.9778	0.3028	0.5352	0.057*	
C10	0.6715 (8)	0.3596 (3)	0.5440 (2)	0.0404 (11)	
C11	0.5015 (10)	0.3997 (4)	0.5088 (3)	0.0508 (13)	
H11	0.3744	0.4202	0.5311	0.061*	
C12	0.5193 (11)	0.4094 (4)	0.4411 (3)	0.0549 (14)	
H12	0.4051	0.4364	0.4171	0.066*	
C13	1.0014 (8)	0.7956 (3)	0.7647 (2)	0.0421 (12)	
C14	0.7877 (8)	0.8049 (3)	0.7237 (2)	0.0401 (12)	
H14	0.7464	0.7489	0.7054	0.048*	
C15	0.5588 (13)	0.9273 (4)	0.7485 (4)	0.0726 (19)	
H15A	0.6620	0.9654	0.7718	0.087*	
H15B	0.4042	0.9442	0.7583	0.087*	
C16	0.8030 (11)	0.8693 (5)	0.6673 (3)	0.0729 (19)	
H16A	0.9432	0.9014	0.6702	0.087*	
H16B	0.7977	0.8404	0.6245	0.087*	
C17	0.6028 (15)	0.9278 (5)	0.6752 (4)	0.094 (3)	
H17A	0.6379	0.9851	0.6596	0.113*	
H17B	0.4729	0.9063	0.6507	0.113*	
N1	0.9739 (9)	0.5403 (3)	0.5517 (3)	0.0627 (13)	
N2	0.7296 (12)	0.3891 (4)	0.3376 (3)	0.0710 (16)	
N3	0.5998 (7)	0.8362 (3)	0.7673 (2)	0.0465 (11)	
O1	1.0691 (9)	0.5029 (3)	0.5975 (3)	0.0833 (15)	
O2	1.0428 (9)	0.5400 (3)	0.4942 (3)	0.0866 (15)	
O3	0.1964 (7)	0.7261 (3)	0.60652 (19)	0.0642 (12)	
H3C	0.1632	0.7234	0.6462	0.096*	
O4	0.8932 (11)	0.3577 (4)	0.3096 (2)	0.0953 (18)	
O5	0.5857 (13)	0.4289 (4)	0.3074 (3)	0.119 (2)	
O6	0.6447 (6)	0.3513 (3)	0.61037 (16)	0.0571 (10)	
H6A	0.7548	0.3265	0.6262	0.086*	
O7	1.1759 (6)	0.7746 (3)	0.73339 (18)	0.0570 (10)	
O8	0.9884 (7)	0.8096 (3)	0.82530 (17)	0.0648 (12)	
H3A	0.653 (11)	0.832 (4)	0.811 (3)	0.064 (17)*	
H3B	0.470 (11)	0.804 (5)	0.760 (4)	0.10 (3)*	

### Atomic displacement parameters (Å^2^)

**Table:**

	*U*^11^	*U*^22^	*U*^33^	*U*^12^	*U*^13^	*U*^23^
C1	0.045 (3)	0.038 (3)	0.057 (3)	−0.005 (2)	0.004 (2)	−0.007 (3)
C2	0.050 (3)	0.046 (3)	0.055 (3)	−0.004 (3)	−0.008 (3)	0.004 (3)
C3	0.048 (3)	0.053 (3)	0.039 (3)	−0.003 (3)	0.002 (2)	0.001 (2)
C4	0.043 (3)	0.050 (3)	0.044 (3)	−0.002 (2)	−0.001 (2)	−0.003 (2)
C5	0.064 (4)	0.064 (4)	0.039 (3)	0.009 (3)	−0.001 (3)	0.001 (3)
C6	0.063 (4)	0.057 (3)	0.045 (3)	−0.001 (3)	0.010 (3)	−0.006 (3)
C7	0.067 (4)	0.046 (3)	0.034 (3)	−0.007 (3)	0.001 (2)	−0.002 (2)
C8	0.051 (3)	0.055 (3)	0.049 (3)	0.002 (3)	0.014 (3)	−0.008 (3)
C9	0.040 (3)	0.054 (3)	0.047 (3)	0.006 (2)	−0.002 (2)	−0.003 (3)
C10	0.037 (3)	0.049 (3)	0.036 (2)	−0.004 (2)	0.000 (2)	−0.004 (2)
C11	0.043 (3)	0.061 (3)	0.049 (3)	0.009 (3)	0.003 (2)	−0.001 (3)
C12	0.056 (4)	0.057 (3)	0.051 (3)	0.008 (3)	−0.013 (3)	0.006 (3)
C13	0.031 (2)	0.055 (3)	0.040 (3)	−0.002 (2)	0.003 (2)	−0.005 (2)
C14	0.028 (2)	0.057 (3)	0.035 (2)	0.001 (2)	0.0025 (19)	−0.005 (2)
C15	0.066 (4)	0.064 (4)	0.087 (5)	0.013 (3)	0.014 (4)	−0.005 (4)
C16	0.058 (4)	0.104 (5)	0.057 (4)	0.017 (4)	0.015 (3)	0.022 (4)
C17	0.076 (5)	0.103 (6)	0.104 (6)	0.025 (4)	0.020 (4)	0.046 (5)
N1	0.051 (3)	0.049 (3)	0.088 (4)	−0.005 (2)	0.006 (3)	−0.012 (3)
N2	0.103 (5)	0.067 (4)	0.042 (3)	−0.013 (3)	0.004 (3)	−0.001 (3)
N3	0.030 (2)	0.068 (3)	0.041 (2)	0.004 (2)	0.0055 (18)	0.002 (2)
O1	0.065 (3)	0.075 (3)	0.110 (4)	0.019 (2)	−0.008 (3)	−0.001 (3)
O2	0.074 (3)	0.092 (3)	0.094 (4)	0.011 (3)	0.028 (3)	−0.012 (3)
O3	0.055 (2)	0.089 (3)	0.048 (2)	0.022 (2)	0.0020 (19)	0.000 (2)
O4	0.135 (5)	0.101 (4)	0.050 (3)	−0.013 (4)	0.030 (3)	−0.009 (3)
O5	0.158 (6)	0.145 (6)	0.052 (3)	0.023 (5)	−0.014 (4)	0.028 (3)
O6	0.042 (2)	0.092 (3)	0.0365 (19)	0.003 (2)	0.0030 (16)	0.0015 (19)
O7	0.0301 (18)	0.089 (3)	0.052 (2)	0.0065 (19)	0.0028 (16)	−0.010 (2)
O8	0.042 (2)	0.113 (4)	0.039 (2)	0.005 (2)	−0.0036 (17)	−0.015 (2)

### Geometric parameters (Å, º)

**Table:**

C1—C6	1.368 (8)	C13—O8	1.237 (6)
C1—C2	1.369 (8)	C13—O7	1.250 (6)
C1—N1	1.461 (8)	C13—C14	1.512 (7)
C2—C3	1.382 (8)	C14—N3	1.493 (6)
C2—H2	0.9300	C14—C16	1.514 (8)
C3—C4	1.385 (7)	C14—H14	0.9800
C3—H3	0.9300	C15—N3	1.491 (8)
C4—O3	1.347 (6)	C15—C17	1.493 (11)
C4—C5	1.380 (7)	C15—H15A	0.9700
C5—C6	1.367 (8)	C15—H15B	0.9700
C5—H5	0.9300	C16—C17	1.502 (10)
C6—H6	0.9300	C16—H16A	0.9700
C7—C8	1.364 (8)	C16—H16B	0.9700
C7—C12	1.377 (8)	C17—H17A	0.9700
C7—N2	1.449 (7)	C17—H17B	0.9700
C8—C9	1.375 (7)	N1—O2	1.223 (7)
C8—H8	0.9300	N1—O1	1.225 (7)
C9—C10	1.385 (7)	N2—O5	1.213 (8)
C9—H9	0.9300	N2—O4	1.219 (8)
C10—O6	1.347 (6)	N3—H3A	0.94 (6)
C10—C11	1.377 (7)	N3—H3B	0.93 (5)
C11—C12	1.370 (7)	O3—H3C	0.8200
C11—H11	0.9300	O6—H6A	0.8200
C12—H12	0.9300		
			
C6—C1—C2	121.9 (5)	N3—C14—C13	109.5 (4)
C6—C1—N1	119.0 (5)	N3—C14—C16	105.3 (4)
C2—C1—N1	119.0 (5)	C13—C14—C16	114.8 (5)
C1—C2—C3	118.8 (5)	N3—C14—H14	109.0
C1—C2—H2	120.6	C13—C14—H14	109.0
C3—C2—H2	120.6	C16—C14—H14	109.0
C2—C3—C4	119.9 (5)	N3—C15—C17	102.9 (5)
C2—C3—H3	120.1	N3—C15—H15A	111.2
C4—C3—H3	120.1	C17—C15—H15A	111.2
O3—C4—C5	118.0 (5)	N3—C15—H15B	111.2
O3—C4—C3	122.3 (5)	C17—C15—H15B	111.2
C5—C4—C3	119.8 (5)	H15A—C15—H15B	109.1
C6—C5—C4	120.4 (5)	C17—C16—C14	106.1 (5)
C6—C5—H5	119.8	C17—C16—H16A	110.5
C4—C5—H5	119.8	C14—C16—H16A	110.5
C5—C6—C1	119.1 (5)	C17—C16—H16B	110.5
C5—C6—H6	120.4	C14—C16—H16B	110.5
C1—C6—H6	120.4	H16A—C16—H16B	108.7
C8—C7—C12	121.6 (5)	C15—C17—C16	103.7 (6)
C8—C7—N2	119.2 (6)	C15—C17—H17A	111.0
C12—C7—N2	119.3 (6)	C16—C17—H17A	111.0
C7—C8—C9	119.7 (5)	C15—C17—H17B	111.0
C7—C8—H8	120.2	C16—C17—H17B	111.0
C9—C8—H8	120.2	H17A—C17—H17B	109.0
C8—C9—C10	119.2 (5)	O2—N1—O1	123.5 (6)
C8—C9—H9	120.4	O2—N1—C1	118.5 (6)
C10—C9—H9	120.4	O1—N1—C1	118.0 (6)
O6—C10—C11	117.6 (5)	O5—N2—O4	122.1 (6)
O6—C10—C9	121.9 (5)	O5—N2—C7	119.5 (6)
C11—C10—C9	120.5 (4)	O4—N2—C7	118.4 (6)
C12—C11—C10	120.1 (5)	C15—N3—C14	106.6 (4)
C12—C11—H11	120.0	C15—N3—H3A	111 (4)
C10—C11—H11	120.0	C14—N3—H3A	106 (4)
C11—C12—C7	119.0 (5)	C15—N3—H3B	110 (5)
C11—C12—H12	120.5	C14—N3—H3B	110 (5)
C7—C12—H12	120.5	H3A—N3—H3B	112 (6)
O8—C13—O7	126.2 (5)	C4—O3—H3C	109.5
O8—C13—C14	117.7 (4)	C10—O6—H6A	109.5
O7—C13—C14	116.1 (4)		
			
C6—C1—C2—C3	0.0 (8)	O8—C13—C14—N3	3.8 (7)
N1—C1—C2—C3	179.4 (5)	O7—C13—C14—N3	−176.0 (5)
C1—C2—C3—C4	−1.4 (8)	O8—C13—C14—C16	121.9 (6)
C2—C3—C4—O3	−178.1 (5)	O7—C13—C14—C16	−57.8 (7)
C2—C3—C4—C5	1.7 (8)	N3—C14—C16—C17	−7.9 (7)
O3—C4—C5—C6	179.0 (5)	C13—C14—C16—C17	−128.4 (6)
C3—C4—C5—C6	−0.8 (8)	N3—C15—C17—C16	−39.1 (8)
C4—C5—C6—C1	−0.5 (9)	C14—C16—C17—C15	29.2 (8)
C2—C1—C6—C5	0.9 (9)	C6—C1—N1—O2	0.4 (8)
N1—C1—C6—C5	−178.5 (5)	C2—C1—N1—O2	−179.0 (5)
C12—C7—C8—C9	−0.7 (9)	C6—C1—N1—O1	−179.3 (5)
N2—C7—C8—C9	178.9 (5)	C2—C1—N1—O1	1.3 (7)
C7—C8—C9—C10	1.0 (8)	C8—C7—N2—O5	−175.9 (6)
C8—C9—C10—O6	179.1 (5)	C12—C7—N2—O5	3.7 (9)
C8—C9—C10—C11	−0.9 (8)	C8—C7—N2—O4	3.9 (8)
O6—C10—C11—C12	−179.6 (5)	C12—C7—N2—O4	−176.5 (6)
C9—C10—C11—C12	0.3 (8)	C17—C15—N3—C14	34.8 (7)
C10—C11—C12—C7	0.1 (9)	C13—C14—N3—C15	107.3 (5)
C8—C7—C12—C11	0.1 (9)	C16—C14—N3—C15	−16.6 (6)
N2—C7—C12—C11	−179.5 (5)		

### Hydrogen-bond geometry (Å, º)

**Table:**

*D*—H···*A*	*D*—H	H···*A*	*D*···*A*	*D*—H···*A*
N3—H3*A*···O8	0.94 (6)	2.03 (6)	2.606 (6)	118 (5)
N3—H3*B*···O7^i^	0.93 (5)	1.87 (6)	2.766 (6)	160 (7)
O3—H3*C*···O7^i^	0.82	1.92	2.656 (5)	148
O6—H6*A*···O8^ii^	0.82	1.82	2.604 (5)	159
C11—H11···O1^i^	0.93	2.59	3.503 (8)	169

Symmetry codes: (i) *x*−1, *y*, *z*; (ii) −*x*+2, *y*−1/2, −*z*+3/2.
